# Virological Surveillance of Influenza Viruses during the 2008–09, 2009–10 and 2010–11 Seasons in Tunisia

**DOI:** 10.1371/journal.pone.0074064

**Published:** 2013-09-19

**Authors:** Awatef El Moussi, Francisco Pozo, Mohamed Ali Ben Hadj Kacem, Juan Ledesma, Maria Teresa Cuevas, Inmaculada Casas, Amine Slim

**Affiliations:** 1 Unit Virology, Microbiology Laboratory, National Influenza Centre, Charles Nicolle's Hospital, Tunis, Tunisia; 2 Influenza and Respiratory Viruses Unit, National Influenza Centre, Centro Nacional de Microbiología, Instituto de Salud Carlos III, Majadahonda, Madrid, Spain; George Mason University, United States of America

## Abstract

**Background:**

The data contribute to a better understanding of the circulation of influenza viruses especially in North-Africa.

**Objective:**

The objective of this surveillance was to detect severe influenza cases, identify their epidemiological and virological characteristics and assess their impact on the healthcare system.

**Method:**

We describe in this report the findings of laboratory-based surveillance of human cases of influenza virus and other respiratory viruses' infection during three seasons in Tunisia.

**Results:**

The 2008–09 winter influenza season is underway in Tunisia, with co-circulation of influenza A/H3N2 (56.25%), influenza A(H1N1) (32.5%), and a few sporadic influenza B viruses (11.25%). In 2010–11 season the circulating strains are predominantly the 2009 pandemic influenza A(H1N1)pdm09 (70%) and influenza B viruses (22%). And sporadic viruses were sub-typed as A/H3N2 and unsubtyped influenza A, 5% and 3%, respectively. Unlike other countries, highest prevalence of influenza B virus Yamagata-like lineage has been reported in Tunisia (76%) localised into the clade B/Bangladesh/3333/2007. In the pandemic year, influenza A(H1N1)pdm09 predominated over other influenza viruses (95%). Amino acid changes D222G and D222E were detected in the HA gene of A(H1N1)pdm09 virus in two severe cases, one fatal case and one mild case out of 50 influenza A(H1N1)pdm09 viruses studied. The most frequently reported respiratory virus other than influenza in three seasons was RSV (45.29%).

**Conclusion:**

This article summarises the surveillance and epidemiology of influenza viruses and other respiratory viruses, showing how rapid improvements in influenza surveillance were feasible by connecting the existing structure in the health care system for patient records to electronic surveillance system for reporting ILI cases.

## Introduction

Identification and characterization of circulating influenza viruses is essential to detect the emergence of antigenic drift variants causing influenza epidemics and novel A subtypes with the potential to cause an influenza pandemic. Thus, virological surveillance of influenza provides a basis for selection of the virus strains to be included in the annual formulation of influenza vaccines [1]. Surveillance data from the African continent has increased substantially in the past five years [2]–[5], but they are still insufficient to allow for a thorough understanding of influenza virus circulation patterns on the continent and their associated morbidity and mortality, or to inform influenza control strategies [6], [7]. The primary objective of this study was to develop or strengthen influenza sentinel surveillance systems in line with WHO standards in selected North African countries. In the past decade, information of epidemic strains from Tunisia was largely unknown due to lack of any systemic study. In this present study we are reporting the activity and circulation of influenza viruses during three seasons (2008–2009, 2009–2010 and 2010–2011) as a part of global influenza surveillance network, which was expanded to Tunisia since 1980.

**Figure 1:**
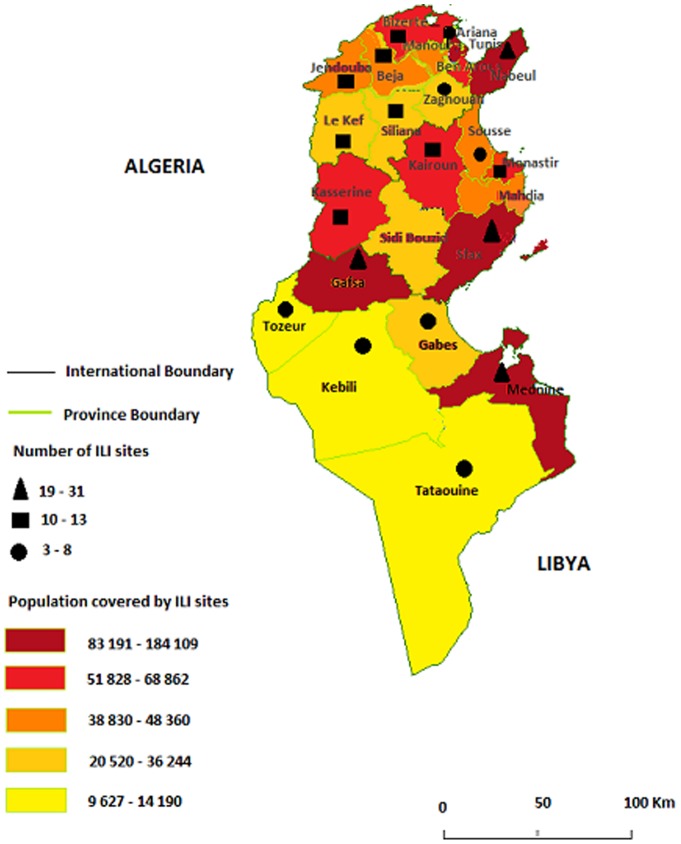
Map of ILI sites and population covered by ILI sites in different Tunisian governorates in 2009–2010 season.

A subset of sentinel primary care physicians participating in virological surveillance schemes in the community submits respiratory samples for virological testing from patients presenting in primary health care with an ILI, as well as all regional emergency centres and hospitals that take on surveillance of influenza from community, hospitalized and fatal cases. The surveillance of influenza and other respiratory viruses is undertaken by 268 primary care centres for adult and pediatric patients (Fig. 1) distributed in 24 governorates covering 2.7% of general Tunisian population (Table 1). Sentinel physicians report weekly the total number of patient visits to their facilities for influenza-like illness (ILI) and acute respiratory infection (ARI) within four age categories (0–4 years, 5–14 years, 15–64 years, and 65+ years). Sentinel physicians are asked to collect respiratory specimens from patients with symptoms of ILI or ARI. ILI was defined as an outpatient with fever (38°C) and cough or sore throat with onset less than five days prior to presentation in the absence of a specific diagnosis. ARI was defined as an outpatient with sudden onset of respiratory signs including cough, difficulty breathing, rhinitis and general symptoms such as fever, headache, fatigue, and myalgia less than five days prior to presentation. The physicians sent specimens for influenza testing and basic demographic data from a subset of patients with ILI each year during October through May to the National Influenza Centre (NIC) situated at the Charles Nicolle's Hospital Tunis, provided that the number of consultants is 10% above the total number of consultants in the sentinel centre. In addition, according to World Health Organization (WHO) recommendations, severe acute respiratory infection (SARI) related to influenza viruses has been added to existing outpatient surveillance systems to fully describe the spectrum of disease related to influenza and to identify individuals at highest risk for severe disease. As a result, various existing routine influenza surveillance systems in Tunisia were enhanced or supplemented to gain a rapid understanding of this novel virus, to monitor its spread and impact, and to evaluate the uptake, impact and effectiveness of the various countermeasures that were implemented.

**Table 1:** Geographic and demographic representation of ILI in Tunisia in 2009–2010 season.

Gouvernourate	Number of regionalsites	Number of ILI sites	Population covered by ILI sites	Total population
Tunis	49	6	122 838	1 003 158
Ariana	25	6	114 726	478 036
Mannouba	40	5	45 405	363 250
Ben Arous	49	6	68 862	562 372
Bizerte	90	10	60 710	546 355
Nabeul	125	31	184 109	742 040
Zaghouan	49	6	20 736	169 351
Beja	94	12	39 288	307 733
Jendouba	114	12	44 700	424 666
El Kef	94	13	36 244	259 276
Siliana	88	10	26 520	236 060
kairouan	130	12	51 828	561 474
Sousse	97	8	48 360	598 450
Mahdia	113	11	38 830	395 360
Monastir	101	10	52 190	501 060
Kasserine	118	12	50 676	430 698
Sfax	156	19	112 480	917 589
Gabes	86	8	31 216	358 980
Kebili	57	5	12 910	149 759
Gafsa	91	23	83 191	336 390
Sidi Bouzid	111	10	33 170	411 290
Tozeur	32	3	9 627	102 703
Mednine	112	24	96 384	453 772
Tataouin	62	6	14 190	146 616
Total	2083	268	341 020	10 456 438

During each season, oro-pharyngeal and naso-pharyngeal swabs were collected from ILI and SARI patients enrolled under the virological surveillance system and placed in viral transport medium (Vircell, Spain®). Oro-pharyngeal and nasopharyngeal swabs collected from the same patient were placed in one cryovial, stored at 4°C at the sentinel site, and transported daily to the NIC. The samples taken from severe cases were from children and/or adult hospitalised in the care unit or patients hospitalized with severe acute respiratory infection were additionally tested for other respiratory viruses. For all participants, respiratory specimens collection were performed after informed consent, under the supervision of local sanitary authorities. It is just an informal statement of the study and members of the ethic committee have previously always approved the work of the NIC. In fact, patients included in this study were of all ages and consulted the sentinel clinics or were hospitalized in state or private hospitals for influenza like symptoms infection. The consent was verbal because the patients, or parents in the case of minors, accept the test for Influenza viruses since it is free and safe. Until now, written consent is judged not necessary by the ethics committee.

Samples were analysed for diagnostic purposes using the real time PCR CDC protocol [8] for detection of influenza viruses and using xTAG® Respiratory Viral Panel Fast (Abbott Molecular, Germany) and the Luminex® technology for other respiratory viruses. All influenza positive samples were sub-typed using specific real-time PCRs for influenza A(H1N1)pdm09, A/H3N2 and for influenza B viruses using “Influenza Virus B Real Time RT-PCR Kit”, “Subtype H1 of Influenza virus A Real Time RT-PCR Kit” and “Subtype H3 of Influenza virus A Real Time RT-PCR Kit”(Shanghai ZJ Bio-Tech Co., Ltd). Every year, we realized the exchange of influenza strains with WHO Collaborating Centre for Influenza in London. In the pandemic year, we succeeded to cultivate some Tunisian strains of influenza A(H1N1)pdm09 and influenza B in National Influenza Centre Madrid Spain. In fact, cell culture virus isolation will be implemented in the National Influenza Centre in Tunisia in the next few years.

A representative number of influenza viruses were genotypically characterized by analysis of the nucleotide sequence of partial haemagglutinin HA1 chain (931 nucleotide residues) and partial neuraminidase (836 nucleotide residues) genes in order to know if circulating viruses were well-matched with vaccine viruses and check for the most frequent amino acid key changes related to neuraminidase inhibitors resistance respectively. All viruses analysed were amplified and sequenced according to the protocol of National Influenza Centre Madrid [9].

Tunisian sequences were aligned with other sequences from reference influenza A viruses available at the NCBI Influenza Virus Resource (http://WWW.ncbi.nih.gov/genomes/FLU/SwineFlu.html) and Global Initiative on Sharing Avian Influenza Data database (http://WWW.platform.gisiad.org) using Clustal W program implemented in MEGA version 4 under default conditions [10]. The nucleotide sequence data reported in this work were deposited in the GenBank nucleotide sequence data-base with accession numbers JN037697 to JN037779.

## Results

### Epidemiological findings

**Figure 2:**
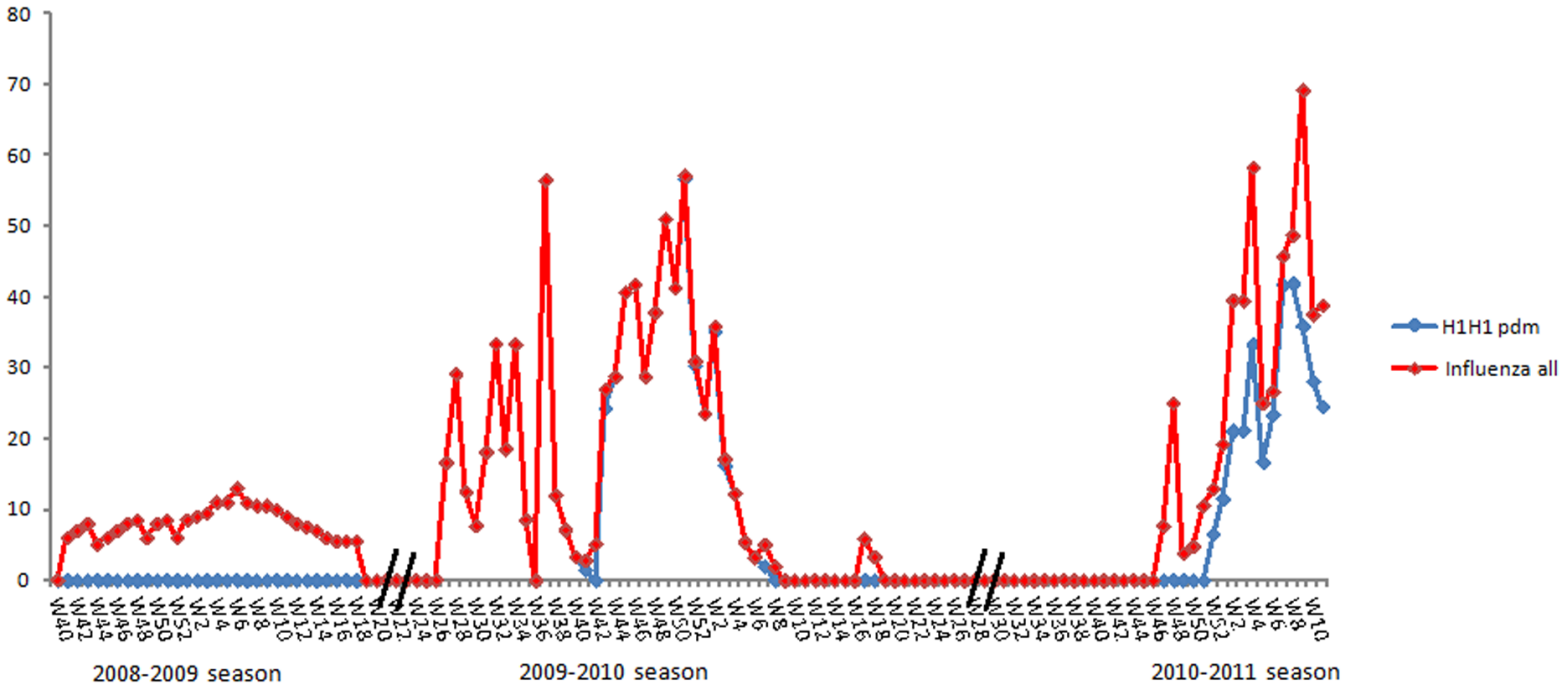
Sentinel data of percentage of specimens positive for influenza in comparison with influenza A(H1N1)pdm09_season 2008–2009, 2009–2010 and 2010–2011.

During the 2008–2009 influenza season, influenza viruses were isolated from week 45/08 (fin August, 2008) to week 16/09 (April, 2009) and peaked during 4 weeks 5–6–7–8/09 (January 27^th^, 2009 and 24 February, 2009) and concerned 67.7% of cases. The number of influenza viruses declined to baseline levels in 28 April 2009 (0%) (Fig. 2). A total of 14 hospitalized confirmed influenza cases were reported and 11 (ranging from 23 days to 6 years old) requiring hospital management in intensive care with severe illness were associated with influenza A/H3N2 infection. Despite the importance of these preliminary results, our surveillance system had limitations in season 2008–2009, and the rate of influenza virus detection remained low (<10%). In fact, the specimen collection and storage techniques may not always have been optimal. In addition, the identification of influenza viruses was performed primarily using immunofluorescence assays which are less sensitive for the detection of influenza viruses than viral culture and real time reverse transcriptase polymerase chain reaction assays (rRT-PCR). The limited number of samples obtained during the 2008–2009 season of surveillance hindered the use of time series analysis to deduce the true number of influenza viruses circulating in Tunisia.

In 2009–2010 pandemic, the first case of pandemic influenza in the country was laboratory confirmed on 18 June 2009. Four confirmed cases were identified in June, but in late July and August (week 28 to 35) an increase in the number of cases was observed. There was a decrease late August and lasted two weeks (Wk 37 and Wk 38). A decrease was observed in week 40 in the number of respiratory samples collected, with a concomitant decrease in the number of laboratory confirmed cases because of period of Aid in which most of industry and schools were closed on holyday. An acceleration of the epidemic was observed in the second week of November, 2009. So the phase of ascent of the epidemic curve lasted 5 weeks, from week 45 to week 49 (from November 17th until December 15^th^, 2009) and concerned 23% of the cases. The epidemic peak spread out over 4 weeks of December (from 49 to week 52, beginning on 30/11/2009 and ending on 27/12/2009), and concerned 60, 6% of all the cases. The peak of the epidemic was reached in the week 37 (56.5%), then in the week 51 (56.8%) of the evolution of the epidemic. The phase of diminution spread out over 5 weeks (from Wk 3 to Wk 7) from 28/12/2009 to 14/2/2010 and concerned 16, 4% of all cases. The pandemic virus has been the predominant strain for the most of the winter season 2009–2010. 1181 admissions to hospital with severe illness have been reported and 170 confirmed cases of influenza A(H1N1)pdm09 with severe infection requiring hospital management in intensive care. A total of 28 pandemic H1N1 influenza-associated deaths were confirmed, and died from a clinically compatible illness or complications attributable to this infection. Six deaths have been reported as pregnant women without particular risk factors. Pandemic influenza was laboratory confirmed in people living in all regions of the country.

Early detections of influenza viruses were first reported during the season 2010–2011 in week 47–2010 (15–21 November) from cases in the community, although influenza A (H1N1)pdm09 was not detected until week 52–2010 (20–26 December). Influenza A(H1N1)pdm09 viruses, followed by influenza B, have been the predominant influenza viruses circulating in the community through the entire season. A prolonged tail to the season was noted, as indicated by continued swab positivity for influenza B. The curve of cases of influenza viruses spread out over 11 weeks (from Wk 1 to Wk 11). There is a decrease of this curve during two weeks (Wk 5 and Wk 6) (in January 24–30^th^ and January 31^st^- February 6^th^). This descent of curve is probably due to the disturbances in the schooling, the transport and the work in this period. Two peaks have been reported in the Wk 4 and Wk 9 (58.3% and 69.2%).We observe the decrease of influenza A (H1N1)pdm09 in the sentinel samples since week 11. Nine influenza-associated deaths were confirmed (ranging 19 to 57 years old) in an ante mortem or post mortem specimen. Five of these fatal cases were pregnant women with an underlying clinical risk factor (mean 32 year old). It is important to note, that all deaths were associated with influenza A(H1N1)pdm09 infection. In fact, pregnancy was identified as a particular risk group for adverse influenza outcome during previous pandemics, and also during seasonal influenza [11], [12].

Two respiratory disease outbreaks in closed settings were reported during the 2010–2011 season; one in the intensive care unit of Charles Nicolle's hospital concerning eight members of the medical staff and the second in the intensive care unit of Rabta's hospital of Tunis when a patient 67 year old diabetic died after infection. Ten deaths were virologically confirmed in two separate outbreaks with influenza A(H1N1)pdm09 detected. It was expected that the virus would behave as a seasonal virus and continue to circulate in the population. Its behaviour, however, could not be reliably predicted [13], although it was considered likely that the virus would continue to cause serious disease in a minority of those infected in younger age groups and people in high-risk groups [14]. Severe cases were defined as any condition or clinical presentation requiring hospital admission for clinical management according to WHO guidance criteria [15]. Severity indicators suggested a higher level of morbidity in terms of the daily number of cases of confirmed or suspected influenza in critical care in pandemic year compared to the 2008–2009 and 2010–2011 seasons. Mortality, in terms of excess deaths and individual fatal cases, was also higher in 2010–2011 than the 2008–2009. Total excess deaths, however, were lower than seen in the 2009–2010 pandemic.

**Figure 3:**
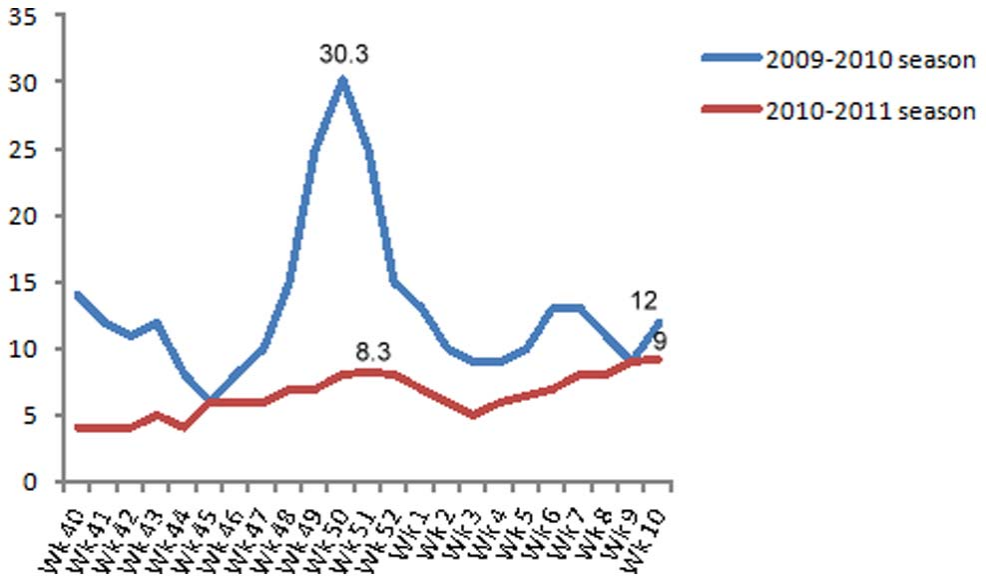
Percentage of consultant in sentinel centre during 2009–2010 and 2010–2011.

Overall, influenza activity in Tunisia in 2010–2011 reached a level higher than that seen in the winter of the 2008–2009 season, but lower than during the first wave of the pandemic in the summer of 2009. In fact, in season 2010–2011 over 50% of sentinel specimens were tested positive for influenza. This intensity of influenza activity was similar to that observed during the peak of the 2009–2010 ‘pandemic’ season. This may reflect a greater intensity of influenza circulation resulting from the introduction of a novel virus into naïve human populations, and/or improvements in the sensitivity of laboratory diagnostic methods to detect influenza in use in Tunisia. However the percentage of consultants for ILI or ARI in the sentinel centres in Tunisia in 2010–2011 season was lower (8.3%) than the percentage of consultants in the pandemic year (30.3%) (Fig. 3). Clinical consultation rates for ILI or/and ARI were declining overall, whereas the percentage of consultants in the next year of the pandemic was not correlated to virological analysis of influenza viruses in Tunisia. Despite the increased number of samples obtained in season 2010–2011 under the enhanced surveillance system, there were limitations to our ILI surveillance system. This is probably due to the participating clinicians may have not correctly identified all ILI or SARI cases. Also it is possible due to a sub-declaration of the clinical services in the sentinel centres in comparison with the last year in which the medical crow was more motivated by the Ministry of Health because of the pandemic. The same situation has been reported in England by WHO European Influenza Network [16]. In fact, both the burden of severe respiratory infection and the proportion due to viral etiologies including influenza are largely undocumented in Africa highlighting the need for continued development of respiratory illness surveillance in this continent [17].

### Virological findings

**Figure 4:**
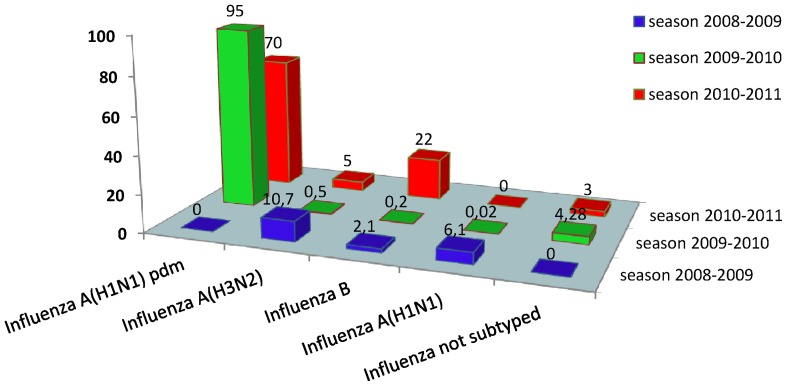
Percentage of influenza viruses in season 2008–2009, 2009–2010 and 2010–2011.

During the 2008–2009 season, 420 specimens were collected from patients at sentinel sites. Of those, 80 (19%) were positive for influenza viruses. 45 influenza A/H3N2 (Perth lineage) (56.25%), 26 A/H1N1(32.5%) and 9 influenza B (11.25%) (Fig. 4). During the pandemic year, a total of 3865 out of 7350 respiratory specimens coming from the sentinel physicians network were positive for influenza. Of these positive specimens, 3836 were sub-typed as influenza A(H1N1)pdm09. In winter 2009–2010, very few specimens tested positive for influenza subtypes other than influenza A(H1N1)pdm09 (95%), influenza B virus 8 (0.1%) (Victoria lineage), 1 (0.02%) formerly seasonal influenza A/H1N1, 20 (0.5%) influenza A/H3N2 (Perth lineage), and 4.28% were influenza A unknown subtype. In 2010–2011 season, 181 out of 894 of the cases were positive for influenza, namely 146 (70%) influenza A(H1N1)pdm09, 33 (22%) influenza B virus (Victoria and Yamagata lineages), 2 (5%) were confirmed as influenza A/H3N2, and 3 (3%) unsubtyped influenza A. We identified a co-circulation of both influenza A(H1N1)pdm09 and influenza type B. But Influenza A(H1N1)pdm09 continue to predominate among circulating influenza viruses in Tunisia. A noticeable difference from the 2009–2010 season has been the relatively high proportion of type B viruses among circulating influenza viruses.

In order to clarify the epidemiological situation of the main agent responsible of respiratory disease in Tunisia in 2008–2009, 2009–2010 and 2010–2011 seasons, other respiratory viruses were identified among sentinel specimens negative for influenza viruses. The identification of these respiratory viruses was undertaken in children admitted to the hospital with acute respiratory infection (Table 2). During season 2008–2009, 148 specimens tested positive for viruses other than influenza including: RSV (70/148; 47.29%), adenovirus (29/148; 19.5%), and Parainfluenza viruses (49/148; 33%). In season 2009–2010, 149 samples were confirmed as respiratory virus. Throughout the year, there are some predominated respiratory viruses: 65 rhinovirus/enterovirus (43.6%), 40 RSVs (26.8%) and 32 hMPVs (21%). In season 2010–2011, a total of 160 specimens were tested positive for respiratory virus and the most frequent respiratory viruses were: RSVs (97/160; 60.6%) and Rhinovirus/enterovirus (37/60; 61.6%). The most common non-flu pathogen circulating in three seasons causing the lower respiratory tract infections leading to hospitalisation especially in children was RSV (207/457; 45.29%). In 2010 in Chile there have been more cases of acute ARI in children but this is attributable to epidemics of respiratory syncytial virus infections (RSV) rather than influenza [18]. This emphasises the importance of countries being able to test for a suite of respiratory pathogens, not just influenza. This data contribute to a better understanding of the circulation of influenza viruses and other respiratory viruses especially in North-Africa.

**Table 2:** Number of respiratory viruses detected by Luminex circulating in Tunisia in 2008–2009, 2009–2010 and 2010–2011 seasons.

		Year	
Respiratory viruses	2008–2009	2009–2010	2010–2011
Adenovirus (Adeno)	29	2	0
Bocavirus (hBoV)	0	5	3
Coronavirus (CoV)	0	2	5
Enterovirus/Rhinovirus	0	65	37
Metapneumovirus (hMPV)	0	32	0
Respiratory syncysial virus (RSV)	70	40	97
Parainfluenza virus 2 (PIV-2)	7	0	1
Parainfluenza virus 3 (PIV-3)	42	0	17
Parainfluenza virus 4 (PIV-4)	0	3	0
Total	148	149	160

**Figure 5:**
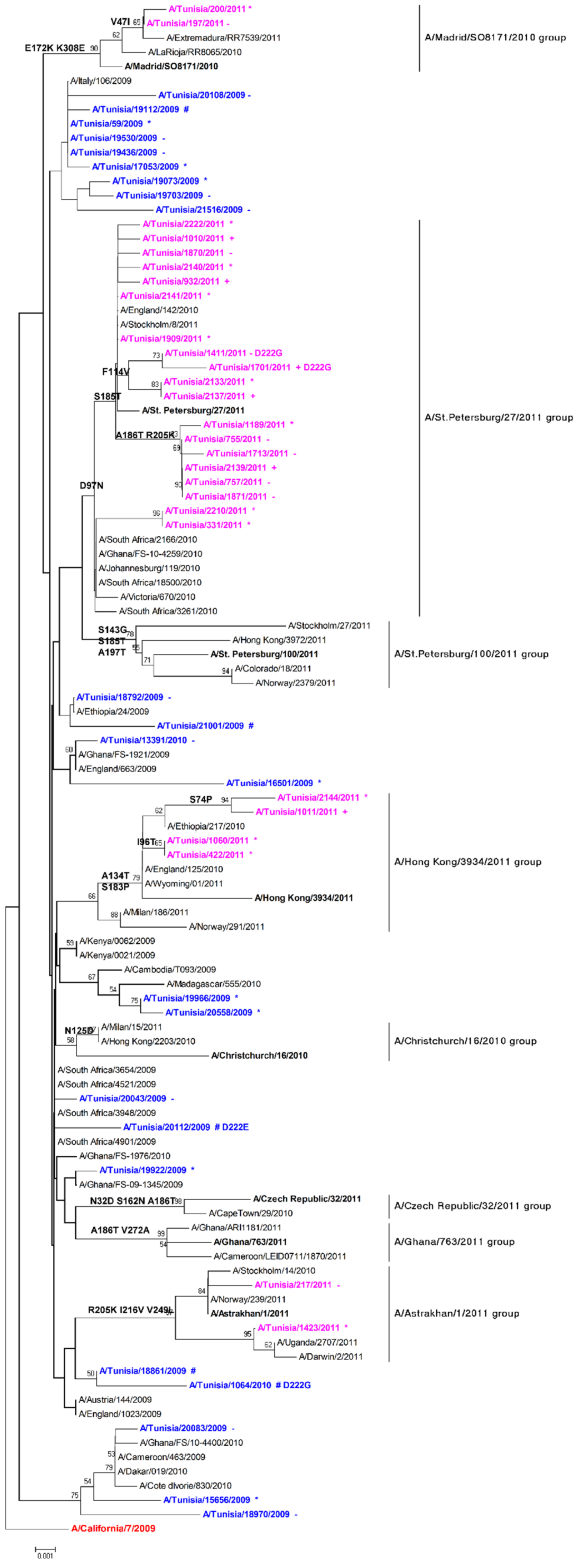
Phylogenetic relationship of partial length HA sequences of influenza A(H1N1)pdm09 viruses from fatal, severe and mild cases in Tunisia during 2009–2010/2010–2011 seasons. Fatal case#; case in care unit+; severe case *; mild case-. The tree was rooted with the vaccine strain A/California/07/2009 (boxed) as outgroup. Branch lengths are drawn to scale. Signature amino acid changes (H1 numbering) are annotated at the nodes of each cluster. Viruses with 222G or 222E changes are marked in the tree.

Phylogenetic analysis of the HA1 nucleotid sequence of 23 influenza A(H1N1)pdm09 viruses from mild, severe (patients hospitalized with severe pneumonia and severe acute respiratory syndrome) and fatal cases, shows that all viruses characterised in Tunisia during season 2009–2010 were outside the seven genetic groups described in the European Centre for Disease Prevention and Control (ECDC) report [19]. A total of 27 HA genes of influenza A(H1N1)pdm09 viruses regardless of whether they were from 2011 from mild, severe cases and from influenza cases admitted to intensive care units were also sequenced and analysed resulting into three groups (Fig. 5). This analysis shows that HA sequences from severe and fatal cases were interspersed with sequences from mild cases in 2009–2010 and 2010–2011 seasons.

All Tunisian 2010 viruses cluster in three main clades: Nineteen isolates gathered from 2010 to 2011 clustered with the HA of clade A/St. Petersburg/27/2011 viruses characterized by D97N and S185T, with additional mutations in minor subclusters such as A186T, K205R and F114V. In A/Astrakhan/1/2011 group we had two strains: A/Tunisia/217/2011 and A/Tunisia/1423/2011 carried D97N, R205K, I216V and V249L substitution. Into this group one strain had an additional mutation H138Q. Third group clustered into A/Hong Kong/3934/2011 clade included four Tunisian strains characterized by A134T and S183P mutations. A separate small cluster of two Tunisian viruses have also been detected (A/Tunisia/197/2011 and A/Tunisia/200/2011) and clustered with Spanish strains with specific amino acid substitutions: V47I, E172K and K308E. Recently, Juan Ledesma et al. have defined and named this group as A/Madrid/SO8189/2010 group [20]. This genetic diversity of Tunisian strains compared to A/California/7/2009 was consistent with expected patterns of virus evolution. Additional substitutions in the position 222 of HA gene were found in 4 Tunisian sequences out of 50 viruses (8%). D222G substitution observed in a total of three viruses analysed (6%). Most of viruses with this mutation were found in severe cases (2/3), one of them from a fatal case [21]. Although most of studies have demonstrated the presence of D222G substitution in severe cases, it was also reported in mild cases [22]–[25]. D222E substitution was found in one out of 50 viruses studied (2%). This sample was taken from a patient with severe clinical syndrome. In fact, D222G mutation has been considered relevant for the acquisition of a hypervirulent phenotype during the 1918 influenza pandemic [26], while the role of D222E in virulence has been ruled out [27]. The clinical significance of D222E mutation is still unclear. Analysis of sequences of neuraminidase gene of influenza A(H1N1)pdm09 from 18 severe cases, not received any antiviral treatment, did not show presence of any of the known mutations associated to neuraminidase inhibitors resistance.

**Figure 6:**
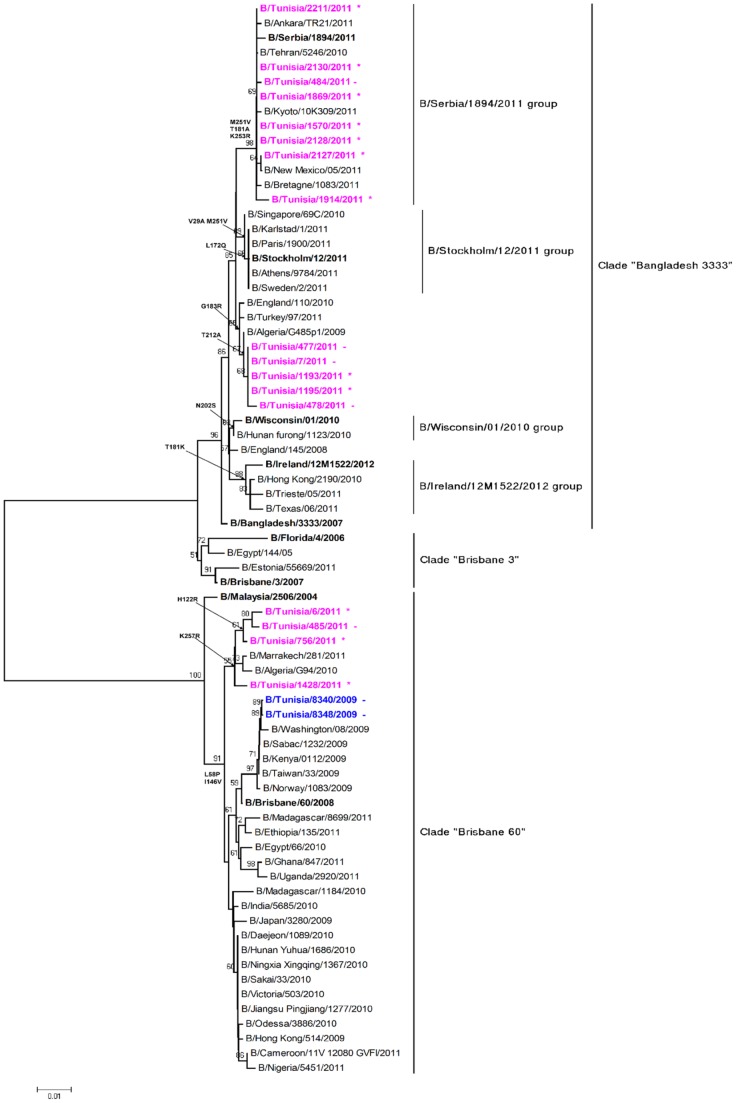
Phylogenetic comparison of partial length HA sequences of influenza B (Yamagata and Victoria-lineage) from severe and mild cases in Tunisia during 2009–2010 and 2010–2011 seasons.

Influenza B viruses were grouped as Victoria lineage or Yamagata lineage on the basis of the HA gene sequence (Fig. 6) showed phylogenetic relationship of HA gene for 19 selected influenza B strains from mild and severe cases, representing 2009–2010 and 2010–2011 seasons, with the concurrent strains of both lineages. Since week 40, 2010 the Tunisian strains belonging to the Yamagata lineage were into the clade B/Bangladesh/3333/2007. Yamagata strains were further divided into two subclusters. The majority of Tunisian strains were localized into B/Serbia/1894/2011. This subcluster of 8 viruses was characterized by three amino acid substitutions M251V, T181A and K253R relative to a previous vaccine strain [19], B/Florida/4/06. A second subcluster contained five viruses defined by amino acid substitutions T121A and G183R. Whereas the strains of Victoria lineage form two subclusters, our study revealed that four of the representative strains from Tunisia, isolated in the year 2011 clustered with B/Victoria/2/87-the oldest strain of Victoria lineage (23.5%). B/Tunisia/6/2011, B/Tunisia/756/2011, B/Tunisia/485/2011 and B/Tunisia/1428/2011 strains showed mutations: L58P and I146V. Two additional substitutions were identified in these strains: H122R and K257R. Co-circulation of both lineages was observed in 2011 as both B/Yamagata/16/88 and B/Victoria/2/87 like strains were observed, whereas in 2009, all strains (B/Tunisia/8340/2009 and B/Tunisia/8348/2009) were phylogenetically similar to the B/Brisbane/2/87 like strains. This study reveals a diverse and complex pattern of influenza B virus strains circulating in Tunisia. Unlike other countries, Tunisian influenza B strains of the Yamagata lineage predominated (13/17; 76%) over those of the Victoria lineage (4%) [28]. Most of Yamagata strains were detected in severe cases (9/13; 69%). Inspite of lower mutation rate and lower pathogenicity than influenza A, influenza B infection contributes to significant proportion of acute respiratory infection among Tunisian people.

## Conclusions

Notably, in season 2008–2009 influenza A/H3N2 viruses, followed by influenza B, have been the predominant influenza viruses circulating in Tunisia. In season 2009–2010, pandemic influenza A(H1N1)pdm09 viruses were the predominant circulating viruses but, in contrast to the 2010–2011 season, there is a higher rate of co-circulation with influenza B viruses. The circulation of other winter viruses such as human respiratory syncytial virus and the particularly cold weather were also identified during three seasons in Tunisia. Unlike the vast majority of influenza B viruses circulating in the world during season 2010–2011, which were from the B/Victoria lineage, most of influenza B Tunisian strains belong to the B-Yamagata lineage, not included in the 2010–2011 vaccine. Therefore a virus strain belonging to the B-Yamagata lineage was indeed recommended for the vaccine composition of the Northern Hemisphere for the season 2012–2013. Appearance of D222G substitution in HA of A(H1N1)pdm09 viruses circulating in Tunisia might be related with severe respiratory disease. Mutations associated with resistance to neuraminidase inhibitors oseltamivir and zanamivir were not detected in the neuraminidase gene. To this end proper surveillance systems should be set up in already existing and well-established national influenza centres to understand the epidemiology of influenza and other respiratory viruses in North Africa, which in turn may help the processes of decision making regarding influenza vaccination on the continent, which may have a high impact on health in Africa.
